# Finding flowers in the dark: nectar-feeding bats integrate olfaction and echolocation while foraging for nectar

**DOI:** 10.1098/rsos.160199

**Published:** 2016-08-10

**Authors:** Tania P. Gonzalez-Terrazas, Carlos Martel, Paulo Milet-Pinheiro, Manfred Ayasse, Elisabeth K. V. Kalko, Marco Tschapka

**Affiliations:** 1 Institute of Evolutionary Ecology and Conservation Genomics, University of Ulm, Albert-Einstein Allee 11, 89069 Ulm, Germany; 2 Smithsonian Tropical Research Institute, Balboa, Panamá, República de Panamá

**Keywords:** acoustic cues, bat pollination, chiropterophily, columnar cactus, floral scent

## Abstract

Nectar-feeding bats depend mainly on floral nectar to fulfil their energetic requirements. Chiropterophilous flowers generally present strong floral scents and provide conspicuous acoustic echoes to attract bats. While floral scents are assumed to attract bats over long distances, acoustic properties of flower structures may provide detailed information, thus supporting the localization of a flower at close ranges. So far, to our knowledge, there is no study trying to understand the relative importance as well as the combination of these generally coupled cues for detection (presence) and localization (exact position) of open flowers in nature. For a better comprehension of the significance of olfaction and echolocation in the foraging behaviour of nectar-feeding bats, we conducted two-choice experiments with *Leptonycteris yerbabuenae*. We tested the bats' behaviour in three experimental scenarios with different cues: (i) olfaction versus echolocation, (ii) echolocation versus echolocation and olfaction, and (iii) olfaction versus echolocation and olfaction. We used the floral scent of the bat-pollinated cactus *Pachycereus pringlei* as olfactory cue and an acrylic paraboloid as acoustic cue. Additionally, we recorded the echolocation behaviour of the bats and analysed the floral scent of *P. pringlei*. When decoupled cues were offered, bats displayed no preference in choice for any of the two cues. However, bats reacted first to and chose more often the coupled cues. All bats echolocated continuously and broadcast a long terminal group before a successful visit. The floral scent bouquet of *P. pringlei* is composed of 20 compounds, some of which (e.g. methyl benzoate) were already reported from chiropterophilous plants. Our investigation demonstrates for the first time to our knowledge, that nectar-feeding bats integrate over different sensory modes for detection and precise localization of open flowers. The combined information from olfactory and acoustic cues allows bats to forage more efficiently.

## 1. Introduction

New World leaf-nosed bats (Phyllostomidae) show a remarkable diversity in food habits [1,2], comprising species feeding on insects, nectar, fruits, blood and small vertebrates [3,4]. Bats perceive their environment using a wide range of sensory modes, including the echolocation system [5]. Over the last decades, research on this sensory system has increased drastically [6,7], improving our understanding of how echolocation works and of the functional and ecological aspects of bat call design [8–10]. Nevertheless, owing to their generally rather stereotyped call parameters (i.e. broadband, frequency-modulated, multi-harmonic, short duration and low-intensity), only few studies focused on the echolocation of the large phyllostomid family [4,11–14].

Recent studies showed that phyllostomids are not as ‘whispering’ as previously thought. Instead they may produce rather loud signals, which are, however, narrowly focused [11,12]. Additionally, they modify their echolocation behaviour to detect and localize their targets [13,15]. In response, e.g. some bat-pollinated plant species evolved structures reflecting conspicuous echoes that attract bats to the food source, such as the vexillum of *Mucuna holtonii* (Fabaceae) flower [16] and the peculiar dish-shaped leaf at *Marcgravia evenia* (Marcgraviaceae) inflorescences [17]. Besides echolocation, phyllostomid bats rely also on further sensory modes during foraging, such as passively listening to acoustic cues, vision and olfaction [18–20]. Scents are known to be used by fruit-eating bats to find ripe fruits [19,21–24]. In an experimental study conducted both in the laboratory and in the field, von Helversen *et al.* [20] tested the attractiveness of 20 scent compounds for the nectarivorous bat *Glossophaga soricina* (Glossophaginae: Phyllostomidae). They found that the animals were selectively attracted by sulfur-containing compounds, in particular by dimethyl disulfide. Floral scent plays a pivotal role in the advertisement of open flowers [25] and may be detected over long distances, yet because scent easily diffuses in air it may provide only low localization accuracy [18]. Therefore, it has been assumed that the main role of olfaction is to guide the bats over larger distances towards areas with available food sources. By contrast, using echolocation, bats can classify and precisely locate an object, and thus, this sensory mode is very important in close-range localization of targets [18,26–28]. Bats may use more than one sensory mode during foraging. For example, insect-eating bats feed mainly on small insects, which have a peak activity just after sunset. Hence, these bats may use a bimodal sensing during foraging; vision for detection of large objects (e.g. trees) and echolocation for searching small prey [29]. Pteropodid bats rely mainly on olfactory cues for detecting ripe fruits; however, vision may play a role in precise localization of the fruit [30]. A recent study shows that the reliance of nectar-feeding bats on certain sensory modes (vision, echolocation or olfaction) during foraging depends strongly on the complexity of the background [31]. Most bat-pollinated flowers present strong floral scents and conspicuous acoustic echoes to attract bats; therefore, we expect that nectar-feeding bats integrate the information of these two sensory modes for efficient detection and localization of open flowers. Vision might also play a role for some nectar-feeding bats, however mainly at crepuscular light or during moonlit nights and especially for finding foraging areas by landmark orientation [32]. As frugivorous and nectar-feeding bats may combine passive (olfaction) and active (echolocation) sensory modes for detecting and locating food, they were recently grouped into a new guild called ‘narrow space passive/active gleaning bats’ [18]. However, so far the relative importance of each cue as well as the combination of both for detection and precise localization of flowers by nectar-feeding bats remains unknown.

In this study, we therefore assessed to what extent nectar-feeding bats rely on olfaction, on echolocation or on a combination of both for detecting and locating food. To elucidate whether the bats reacted differently to olfactory and acoustic cues (singly or combined), we conducted two-choice experiments with *Leptonycteris yerbabuenae* (Phyllostomidae: Glossophaginae). We hypothesized that olfactory cues are used mainly to attract bats into the larger feeding area, while echolocation facilitates precise localization of floral targets at close range. As bat-pollinated flowers usually present acoustic and olfactory cues together [20,26], we predicted that the simultaneous presence of both cues will help to optimize foraging. Besides, we expected that bats will not adapt their echolocation behaviour during the approach to a plain olfactory cue, as no echo-reflecting object suggests acoustically the presence of a potential food source. Conversely, because the acoustic cue used in the experiments presents similar echo-acoustic characteristics to bat-pollinated flowers, when the bats visit the acoustic cue (combined or alone), we expect they will present a similar echolocation behaviour as with the real flower (i.e. more calls per group while approaching and long terminal group before the visit) [15].

## 2. Material and methods

### 2.1. Study species

*Leptonycteris yerbabuenae* is a large (body mass *ca.* 24 g) nectar-feeding bat occurring mainly in arid and semi-arid areas (e.g. tropical dry forest and deserts) from the southwestern United States to Honduras [33]. It is one of the main pollinators of chiropterophilous plants in dry ecosystems, especially of columnar cacti and *Agave* (Agavaceae) [34–36]. We captured bats using mist nets at the exit of a small cave near Kino Bay in the Mexican Sonoran Desert. The study was conducted between April and May 2011, a period when flowers of the columnar cactus *Pachycereus pringlei* (S. Watson) Britton & Rose (Cactaceae) are the main food source for the bats. During the night prior to the experiments, the bats were allowed to habituate in a small room (3 × 4 × 2 m) with ad libitum access to food (17% sugar solution, Nectar-Plus, Nekton®). Each bat was kept in captivity for no longer than four nights and released after the experiments unharmed near the place of capture. We followed the guidelines for the use of wild mammal species in research as recommended by the American Society of Mammalogists [37]. Captures were carried out under permission of the Secretaría de Medio Ambiente y Recursos Naturales, Mexico.

### 2.2. Behavioural experiments and sensory cues

**Figure 1.**
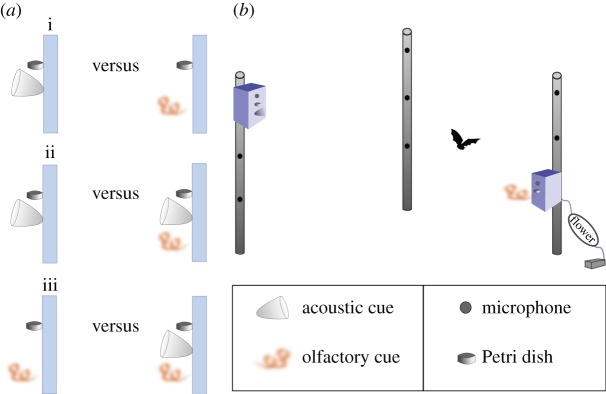
(*a*) Design of the two-choice experiments conducted in this study: (i) acoustic versus olfactory, (ii) acoustic versus acoustic and olfactory, and (iii) olfactory versus acoustic and olfactory cues. Three different trials were conducted per bat. The blue bar represents the front of the sound-absorbing Basotect boxes where the cues and the reward were placed. In all trials, we offered sugar water in a small Petri dish. (*b*) Exemplary set-up for the behavioural experiments. This particular scenario represents the trial acoustic cue versus olfactory cue. Each PVC tube offered three possible positions to present cues. The illustration shows the two boxes in positions 1 (acoustic) and 9 (olfactory).

To establish the relative importance of echolocation and olfaction as well as the combination of both for flower detection and for localization, we conducted two-choice experiments with 15 non-reproductive females of *L. yerbabuenae*. We performed three different trials per bat: (i) olfaction versus echolocation (figure 1*a*), (ii) echolocation versus echolocation and olfaction (figure 1*b*), and (iii) olfaction versus echolocation and olfaction (figure 1*c*).

For providing olfactory cues, we used newly open flowers from several *P. pringlei* plants near the cave. The flower was enclosed in a polyester oven bag of approximately 15 × 10 cm (Toppits®, Germany) that on one side was connected to a membrane pump (G12/01 EB, Rietschle Thomas, Puchheim, Germany) blowing air through the bag at a rate of 200 ml min^−1^. The other side of the bag was connected to a silicone tube used to direct the flow of scented air necessary for the experiments (figure 1*b*). We used one open flower for 2–3 trials, after that we replaced it with a new flower. The acoustic cue consisted of a scentless acrylic paraboloid with a 5 cm diameter and a depth of 5.5 cm. For the combination of both cues, we presented both the floral scent and paraboloid close to each other and at the same time (figure 1*a*).

To avoid unwanted sound reflections, we used boxes of sound-absorbent foam (Basotect®) for offering the different cues to the bats. All boxes had the same size (30 × 20 × 15 cm) and only the sensory cue presented changed between different trials. Each box had one hole where an ultrasound microphone was always present and two possible positions to locate the silicon tube that delivered the scented air and the acrylic paraboloid (figure 1*b*). In all trials, we used a small Petri dish filled with sugar water (17%), which was placed just below the ultrasound microphone (figure 1*b*).

We performed the behavioural experiment in a hexagonal flight cage (4 × 4 × 2.5 m). We placed three PVC tubes (height 1.80 cm) near to the corners within the flight cage. Each of these three tubes offered three locations where the boxes with the sensory cues could be placed (per tube at 60, 120 and 180 cm height), resulting in a total of nine potential positions (figure 1*b*). To avoid spatial learning, the position of the boxes was changed randomly after each trial, excluding the two positions used in the previous trial. We worked with one bat at a time and performed the three trials in a row; the order of trials per bat was also randomized. Bats were exposed only once to each trial. The trial ended either when the bat visited (touched) the Petri dish or when it inserted its snout into the paraboloid. Experiments were performed between 19.00 and 02.00 h in complete darkness. In case the bat did not respond to any of the cues, the trial was ended after 30 min. We recorded the behaviour of the bats with three infrared video cameras (Sony Handycam, Japan). One of the cameras had a fish-eye lens providing a general overview of the entire flight cage. The other two cameras were directed each to one of the boxes to provide a more detailed view of the behaviour of the bats while approaching the different cues. Additionally, we recorded the echolocation behaviour of the bats with two synchronized bat recorders (UltraSoundGate 116Hm, with CM16 microphone capsule and CMPA preamplifier unit, Avisoft Bioacoustics). Every time a bat inspected, approached or visited one of the boxes, a recording was triggered, ranging from 2 s before to 2 s after the trigger event.

### 2.3. Behavioural and acoustic analysis

Using the video recordings, we observed (i) to which cue the bat reacted first and (ii) which cue it finally visited (choice). We scored a successful visit when a bat inserted its snout into the paraboloid or when it touched the small dish with its snout or tongue. In case a bat did not actually visit a cue, we counted the most often approached cue as the choice. To test for differences between the number of first reactions and choices between the paired cues, we conducted a binomial test using SPSS Statistics v. 17.0 (SPSS, IBM). Furthermore, we scored a reaction when the bats flew by with clear interest towards the cue (i.e. pointing the head towards the cue; inspection) or when the bat approached and hovered in front of the cue (approach). To test for differences in the mean number of reactions (inspections and approaches pooled) between the two offered cues, we conducted a non-parametric, Mann–Whitney rank sum test using SigmaSTAT v. 3.5 (Systat Software).

For a first assessment of the echolocation behaviour of the bats while approaching the different cues, we used the software SasLab Pro (Avisoft, Germany). For a more detailed analysis of the long terminal group, we used a custom-made analysis program (Selena, University of Tübingen, Germany), using a Hamming window with a 512 fast Fourier transform and a dynamic range of 60 dB (resolution: 938 Hz 0.066 ms^−1^). To test for significant differences in the ‘terminal group’ parameters between targets, we performed *t*-tests using SigmaSTAT v. 3.5 (Systat Software).

### 2.4. Flower scent sampling and chemical analysis

Floral scent samples (*n* = 8) of *P*. *pringlei* were collected *in situ* using dynamic headspace techniques [38]. Single intact flowers were enclosed in polyester oven bags (Toppits®, Germany). Volatiles were collected during 5 h (from 19.00 to 24.00 h) in an adsorbent tube, through which air from the polyester bag with the flower was drawn at a rate of 200 ml min^−1^ using a membrane pump. Air drawn into the bag was cleaned of atmospheric impurities by a charcoal filter (Orbo-32, Supelco). The absorbent tubes consisted of a glass vial (length 10 cm and inner diameter 4 mm) filled with 5 mg of Super Q (Waters Division of Millipore). Control samples with just ambient air were collected in parallel using the same method as described above. The volatiles trapped in the adsorbent tube were subsequently eluted with 150 ml of pentane (99.9%, Sigma-Aldrich) and stored at −20°C.

To identify the volatiles in the floral scent bouquet of *P*. *pringlei*, we analysed the headspace samples using a mass spectrometer (Quadrupole 5973 Mass Selective Detector; Hewlett-Packard Company, Wilmington, DE, USA) coupled to an Agilent gas chromatograph (HP 6890 Series) fitted with a BPX5 fused-silica capillary column (25 m × 0.22 mm i.d., 0.25 mm film, SGE Inc., Melbourne, Australia). One microlitre of each sample was injected splitless at 50°C. After 1 min, the split valve was opened and the temperature increased by 4°C min^−1^ to 310°C. The injector was set at 310°C. Hydrogen was used as carrier gas at a constant flow of 2.0 ml min^−1^. Data were processed and analysed on the MSD ChemStation Software (Agilent Technologies, Germany). Structure elucidation of individual compounds was performed comparing the mass spectra from our samples with those from commercial libraries (NIST library, ADAMS, library of the Institute of Evolutionary Ecology and Conservation Genomics, Ulm University) and with the spectra of synthetic compounds. To determine the relative ratio of the compounds in the floral scent bouquet of *P. pringlei*, the headspace samples were analysed on an Agilent gas chromatograph (HP 7890 A Series) equipped with a DB-5 capillary column (30 m × 0.25 mm i.d., J&W), and a flame ionization detector (FID), using an analogous procedure as above. Resultant chromatograms were processed and analysed in ChemStation software (Agilent Technologies, Germany).

## 3. Results

### 3.1. Behavioural experiments

**Figure 2.**
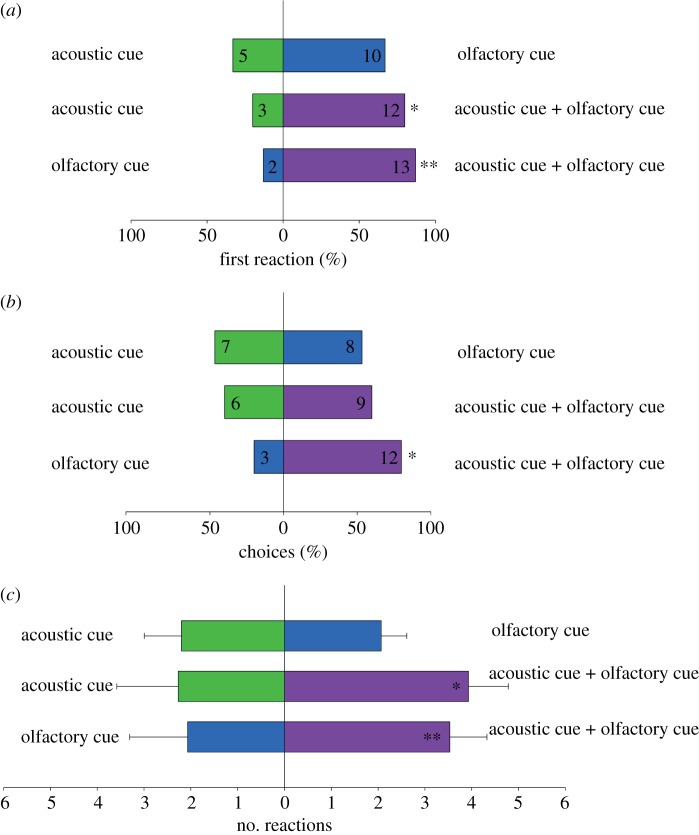
(*a*) First reactions of *Leptonycteris yerbabuenae* to acoustic and olfactory cues (alone or in combination). (*b*) Choices of *L. yerbabuenae* in response to acoustic and olfactory cues (alone or in combination). Differences in behavioural responses for the two-choice experiments were assessed using a binominal test, *p < 0.05, **p < 0.01. The numbers in the bars indicate *n* of bats. (*c*) Number of reactions (approaches + inspections) per bat (*n* = 15) to each of the cues in the two-choice experiments (mean ± s.d.). The bats reacted significantly more frequently to the combined cues (*p < 0.05, **p < 0.01).

In the two-choice experiments, the first reaction of the bats was clearly towards the combination of olfactory and acoustic cues (figure 2*a*). When combined cues were offered against either the acoustic or olfactory cue alone, 80% (*n* = 12) and 87% (*n* = 13) of the bats, respectively, reacted to the combined cues first (binomial test: *p* = 0.035 and *p* = 0.007, respectively). When olfactory and acoustic cues were presented against each other, we found no significant difference in the first reaction of the bats (binomial test: *p* = 0.302). Nevertheless, 10 of the 15 bats (67%) reacted first to the olfactory cue.

We also observed a higher number of choices at combined than at single cues (figure 2*b*). When combined cues were offered against the acoustic cue, we found no significant difference (binomial test: *p* = 0.581). In this case, only 60% (*n* = 9) of the bats visited the combined cues and 40% (*n* = 6) the acoustic cue alone. In almost all cases (80%), when the paraboloid was presented, either combined with the olfactory cue or alone, the bats inserted their head inside the paraboloid trying to extract nectar. However, when combined cues were offered against just olfactory cues, 80% of the bats chose the combined cues (binomial test: *p* = 0.035). As for the first reactions, we found no significant difference in the choice of bats when decoupled acoustic and olfactory cues were offered against each other; 53% of the bats chose the olfactory cue and 47% the acoustic cue (binomial test: *p* = 1) (figure 2*b*).

Finally, when considering the mean number of reactions per bat (inspections and approaches pooled, *n* = 15), bats reacted significantly more often to the combined cues than to either cue alone (Mann–Whitney rank sum test, acoustic and olfactory cues versus acoustic cue: *p* = 0.020, acoustic and olfactory cues versus olfactory cue: *p* = 0.008) (figure 2*c*). In the two-choice experiment where we presented the decoupled cues against each other, the bats showed a similar number of reactions to olfactory (48%) and acoustic cue (52%) (Mann–Whitney rank sum test, acoustic versus olfactory cue: *p* = 0.717).

### 3.2. Echolocation behaviour

**Figure 3.**
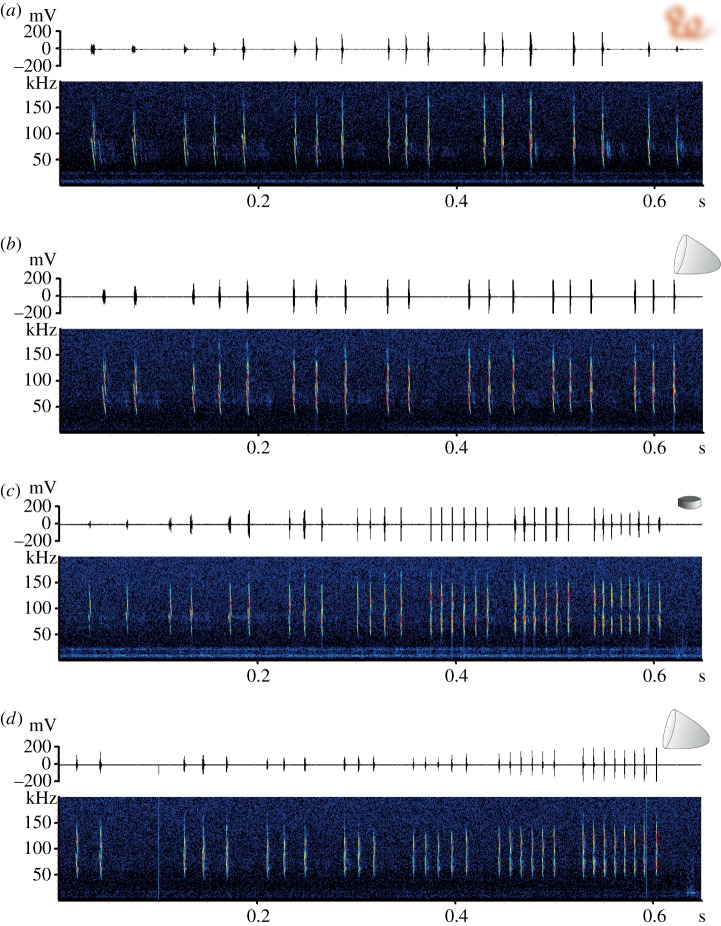
Echolocation behaviour *of Leptonycteris yerbabuenae* when approaching olfactory (*a*) and acoustic cues (*b*). In both cases, the bats emitted groups of 2–3 calls until the end of the approach. When the bats actually visited the small Petri dish (*c*) or the paraboloid (*d*), we observed a similar echolocation behaviour: in both cases they increased the number of calls per group during the approach and produced a long group of calls before inserting their head into the paraboloid or touching the Petri dish.

Independent of the type of cue presented, all bats were continuously echolocating during inspection and approaching flights. The bats showed very similar echolocation behaviour when approaching the olfactory cue and when they approached without visiting the target. Bats continuously broadcasted groups of 2–3 calls during the whole approach (figure 3*a,b*). When only olfactory cues were presented, the bats approached the location where the floral scent was emanating from several times, while always showing the echolocation behaviour described above. However, after some initial inspections and approaches they widened their exploratory behaviour and investigated the box until they successfully visited the nectar dish. The bats presented the same echolocation behaviour when they performed a successful visit no matter to which of the targets (paraboloid, alone or combined with scent, and even when they extracted nectar from the Petri dish). In all cases, they emitted a long terminal group before touching the target (figure 3*c*,*d*). We did not find any significant difference in the call parameters of the long terminal group emitted when visiting the paraboloid or the small dish, respectively (table 1).

**Table 1.** Echolocation call parameters of the long group of calls emitted before visiting the paraboloid or the small Petri dish used as a reward in all experiments.

	terminal group		
	paraboloid	Petri dish	*p*-value
peak frequency (kHz)	68.4 ± 7.4	70.9 ± 6.4	0.342
pulse duration (ms)	1.3 ± 0.3	1.3 ± 0.2	0.935
pulse interval (ms)	11.0 ± 1.2	11.0 ± 1.7	0.935
number of calls	7.6 ± 2.8	8.0 ± 1.8	0.628

### 3.3. Flower scent

Twenty floral scent compounds, belonging to five compound classes (as categorized by Knudsen *et al.* [39]), were identified in the headspace samples (*n* = 8) of *P. pringlei* flowers (table 2). Benzenoids were the most diverse class with eight compounds, followed by aliphatics (6), terpenoids (4), a sulfur-containing compound (1) and a cyclic compound (1).

**Table 2.** Mean relative amount of floral scent compounds of *Pachycereus pringlei*. (Volatiles are grouped in compound classes and listed according to retention index (RI). Tr, trace amount (<0.05%).)

compounds	RI	relative proportion (mean ± s.d.%)
*aliphatics*		
methyl hexanoate	927.50	0.14 ± 0.29
ethyl tiglate	943.49	0.076 ± 0.22
undecane	1100.00	0.77 ± 2.18
ethyl hexanoate	1000.16	1.45 ± 2.75
nonanal	1105.57	1.68 ± 2.15
decanal	1207.55	0.15 ± 0.29
*benzenoids*		
styrene	899.43	0.29 ± 0.82
benzaldehyde	970.85	0.93 ± 1.36
α-methylstyrene	986.60	0.20 ± 0.56
benzyl alcohol	1038.41	0.24 ± 0.46
acetophenone	1070.17	2.05 ± 5.47
2,6-dimethylbenzyl alcohol	1088.38	0.19 ± 0.54
methyl benzoate	1096.10	72.38 ± 4.10
ethyl benzoate	1173.99	16.36 ± 7.04
*cyclic compounds*		
3,3,5-trimethylcyclohexanone	1041.63	2.49 ± 4.20
*terpenoids*		
ethyl 2-methylbutanoate	866.80	0.24 ± 0.68
α-pinene	940.47	0.14 ± 0.40
sulcatone	991.67	0.07 ± 0.19
limonene	1033.74	0.15 ± 0.43
*sulfur-containing compounds*		
dimethyl disulfide^a^	—	Tr

^a^Dimethyl disulfide retention index is not included, because it was close to the elusion peak and RI could not be calculated.

With a relative amount of 72.4%, methyl benzoate was the major compound in the scent bouquet followed by ethyl benzoate with 16.4%. All other volatiles were present in amounts of less than 5%.

## 4. Discussion

### 4.1. Behavioural experiments

Studies investigating the role of floral cues in foraging behaviour of nectar-feeding bats are scarce and focused either on the role of echolocation or on selected floral scent compounds [17,20,26], while the combination of these mostly coupled cues in nature was never investigated. The reactions of the bats in our two-choice experiments allowed us to assess not only the relative importance of either acoustic or olfactory floral cues on bat foraging behaviour, but also their combined effect. The observation that combined cues are more effective in triggering responses than decoupled acoustic and olfactory cues shows that the bats integrate over different sensory modes during foraging.

The first reaction of the bats occurred significantly more often towards the combined cues. When decoupled cues (acoustic versus olfactory cues) were offered against each other, bats did not show any significant preference, although we observed a tendency of the bats to respond first to the olfactory cues (67%). Floral scent compounds are assumed to be used as a primary cue and long distance attractant for nectar and fruit-eating bats, both in the Neo- and the Palaeotropics [18,20,30,40]. The fact that bats tended to react first when the olfactory cue was present (either singly or in combination with the acoustic cue) indicates that scent plays an important role in attracting bats towards the open flower. Nevertheless, some of the bats that reacted first to the scent ended up visiting the acoustic cue. When combined cues were presented against the decoupled acoustic cue, bats reacted consistently first to the combined cues (80%). However, after this first reaction, they kept on investigating their environment and some individuals ended up visiting the scentless paraboloid. This might indicate that the bats initially detected the presence of an open flower through olfaction and that the acoustic cues alone can guide the bats towards the flower opening. Using echolocation, the bats can gather much more precise information on an object structure than using olfactory cues [18]. The fact that bats which previously had never been exposed to the scentless paraboloid inserted their snout without any training demonstrates impressively that bats can localize a flower opening solely by echolocation.

All bats used during the experiments were captured in the Sonoran desert where the main food resources were the flowers of *P. pringlei*. While foraging, bats use an ‘acoustic search image’ that they associate with a potential food source [13,41]. The paraboloid used in the experiments had a similar shape and size as such cactus flowers and the spectral composition of the echo reflected towards the echolocating bat are similar to those of some bat-pollinated plants [26]. In trials where only an acoustic cue was presented, bats were successfully locating and visiting the scentless flower-shaped target. In a previous study with *L. yerbabuenae*, the bats never visited a different artificial target presented during these experiments, an acrylic hemisphere [15], which indicates that the acoustic characteristics of the paraboloid are more similar to the echoes of a natural flower than those of the hemisphere. Another study demonstrated that with the presence of an acoustic signal (i.e. dish-shaped leaves) nectar-feeding bats could detect scentless targets and reduced foraging time [17]. Some bats also chose the olfactory cue even though there was no conspicuous acoustic cue present. In all cases, the bats approached first and most often the small opening where scent was emanating from and only after realizing that there was no acoustic cue, indicating the presence of a potential nectar source, they started also investigating other areas. Contrary to our hypothesis, bats were able to detect and localize targets that presented characteristic olfactory cues and inconspicuous acoustic cues, which indicate that floral scent has also a function as a close-range marker. Floral scent is not only emitted from floral tissues, but there are also some plants that present scented nectar [42]. Scented nectar provides an honest signal to a nectar-feeding animal and thus facilitates the detection and efficient foraging on the actual floral resources, particularly when the composition of the nectar scent presents qualitative or quantitative differences to the overall floral scent [42]. For instance, in flowers of *Agave palmeri*, which is mainly pollinated by bats, dimethyl disulfide is emitted in a higher percentage by the nectar than by the floral tissues [42]. Glossophagine bats are innately attracted to dimethyl disulfide, which is a generally uncommon floral scent compound but is found in several bat-pollinated plants [20,43–46]. Further studies combining chemical analyses of both nectar and floral scent composition with behavioural assays are needed to test the hypothesis that bats distinguish between these two scent bouquets to discriminate empty flowers from those with nectar.

### 4.2. Echolocation behaviour

Bats echolocated continuously during the experiments, irrespective of the presented cue. Echolocation behaviour was very similar when the bats approached the olfactory cue, and when they approached without visiting the acoustic cue, they broadcast groups of 2–4 calls during the entire approach sequence. This suggests that in both situations the bats did not receive the information associated with a food source. However, bats successfully visited the paraboloid, where in some cases they even introduced their head into this artificial target. The spectral composition of the echoes changes with the angle of incidence and thus provides crucial information for the bats that facilitates finding the flower opening [26]. The fact that bats were successfully visiting the acrylic paraboloid confirms that the acoustic characteristics of the paraboloid were perceived by the bats as being similar to those of the natural cactus flowers. However, the bats also visited the Petri dish with nectar that we used as a reward in all experiments. An experimental exclusion of all acoustic cues is very challenging. The small dish provided only weak reflections that are very different from those bats experience in their natural habitat. However, even if the bats have never been exposed before to this artificial object, they visit it successfully. When visiting the Petri dish and the paraboloid, bats emitted a long terminal group of 8 ± 2 (mean ± s.d.) calls before touching the target. Similar behaviour was observed in flight cage experiments with *L. yerbabuenae* while visiting the flowers of *P. pringlei* [15]. This increase in information flow indicates that bats used echolocation for the precise localization of the flower and the flower opening. The fact that bats successfully visit both targets, and that all bats broadcasted this long terminal group before the visit, indicates an important role of echolocation for the localization of food sources. The lack of adaptation of the long terminal group to the respective task suggests that the emission of the terminal group is a stereotypic behaviour shaped more by physiological constraints than by the actual target. Further studies will help to understand the flexibility and function of this long terminal call group emitted by *L. yerbabuenae* just before visiting a flower.

### 4.3. Flower scent

Several groups of unrelated Neotropical plant species have evolved similar floral scents to attract bats [20,39,43–46]. The floral odour bouquet of *P*. *pringlei*, similar to other bat-pollinated cacti, is dominated by benzenoid and phenylpropanoid, and aliphatic compounds [43–45]. However, we found some differences in terms of relative amount of compounds in the scent bouquet. The main volatile of *P*. *pringlei* flowers, i.e. methyl benzoate, has been found previously in other bat-pollinated cacti (e.g. *Pilosocereus pachycladus*), however, at much lower relative amounts [44]. In the literature, there is some evidence that mammals (rats and mice) respond to methyl benzoate and are sensible to changes in concentration [47]. Unfortunately, the significance of this compound for attracting bats to flowers has, to our knowledge, never been investigated in behavioural experiments, making any conclusion in this context premature. Benzaldehyde is the most common aromatic scent compound found in bat-pollinated plants [39] but is also a common floral scent compound recorded in about 2/3 of all flowering plant families [48]. We found only small quantities of dimethyl disulfide in the scent bouquet of *P. pringlei*. New World nectar-feeding bats are particularly attracted to sulfur-containing compounds such as dimethyl disulfide [20], present in the odour bouquet of flowers from several unrelated chiropterophilous plant families, including Cactaceae. However, other bat-pollinated plants that also emit a pungent odour produce only trace amounts of this substance, such as *Parmentiera alata* (Bignoniaceae) [43] or produce no sulfur compounds at all, e.g. the epiphytic rainforest cactus *Weberocereus tunilla* [43,49]. Sulfur-containing compounds are highly volatile and thus are easily lost or overlooked during headspace sampling if they occur only in small quantities. This could explain, at least in part, why we found dimethyl disulfide only in trace amounts in the desert-dwelling flowers of *P*. *pringlei*. Flowers of *A. palmeri* present dimethyl disulfide mainly in the nectar; further studies are needed to assess whether *P. pringlei* emits this compound from the nectar and, if so, whether it is emitted in higher quantities in the nectar than in the floral tissue. The other compounds identified in the floral scent of *P. pringlei* have already been recorded in cacti and other bat-pollinated plants [39,50].

## 5. Conclusion

Our study shows for the first time, to our knowledge, that nectar-feeding bats integrate over different sensory modes (i.e. olfaction and echolocation) to detect and precisely locate flowers. We provide evidence that, contrary to previous knowledge, bats use olfactory cues not only as a long-distance attractant, but also as close-range markers of flowers and suggest that they may differentiate nectar filled from empty flowers using scents. We confirm that acoustic cues play a major role for nectar-feeding bats in target localization and for obtaining detailed information about where to insert the head in search of nectar. The integration of the information received from these two sensory modes permits an increase in foraging efficiency for the nectar-feeding bats.

## Supplementary Material

Supplementary data_cues: Raw data of the flight cage experiments.

## Supplementary Material

Supplementary data_TG: Call parameters of the terminal group per bat.

## Data accessibility

The dataset supporting this article has been uploaded as part of the electronic supplementary material.
